# Supplementary material for: Rapid turnover of life-cycle-related genes in the brown algae
Source: Genome Biol. 2019 Feb 14;20:35. doi: 10.1186/s13059-019-1630-6 (PMC6374913; doi:10.1186/s13059-019-1630-6)
Supplement: Supplementary file 3 — Protein alignments, corresponding Gblocks html file and CDS sequences for the lineage conserved gametophyte-biased genes. (ZIP 2109 kb) [file 13059_2019_1630_MOESM3_ESM.zip › Con_GA_alignments/Ec-12_003690.aa_ali.fasta-gb.htm]

Ec-12\_003690.aa.fasta.trans.aa\_ali.fasta


## Gblocks 0.91b Results

Processed file: **Ec-12\_003690.aa.fasta.trans.aa\_ali.fasta**  
Number of sequences: **2**  
Alignment assumed to be: **Protein**  
New number of positions: **451** (selected positions are underlined in blue)

```
                         10        20        30        40        50        60
                 =========+=========+=========+=========+=========+=========+
Ec-12_003690     MLLLDISPVSEREPYRRAGSRELLADCSQKDKDHPLVSNITRSTGQDLDLPTKSESSQSG
MSTRG.51532.1    ---------------XRMG-RAVAAVPTQQTQ------------------PASPAASTS-
                                                                             


                         70        80        90       100       110       120
                 =========+=========+=========+=========+=========+=========+
Ec-12_003690     CLHTILFRRGVEGSGGYEMNLCRKQIAVSKATHRMAWSFCILGVWCATWEVGRQWGSLKA
MSTRG.51532.1    -------------------------WAVEKDANRTAS-----------------------
                                           ##########                        


                        130       140       150       160       170       180
                 =========+=========+=========+=========+=========+=========+
Ec-12_003690     MHTASRKAATQEGLNCGSRQFPCVESEVRVGRVEVVGPVEKPQSAPLLVSPLLSGEDKGG
MSTRG.51532.1    -----------ERLPVSARNVPISGREIGVSKADLP------------------------
                            ###################                              


                        190       200       210       220       230       240
                 =========+=========+=========+=========+=========+=========+
Ec-12_003690     RKPSAERLPVAEDGTPAGEPSTLSAEGDTDRLHPDLFWKAYAADRYSLLTRGVVVGMEDK
MSTRG.51532.1    --TDGPRIPI--------QDTAIDAEG-TDKLHPDRFWRAYAADRYSLLTRGVVAGNGDL
                                             ############################### 


                        250       260       270       280       290       300
                 =========+=========+=========+=========+=========+=========+
Ec-12_003690     --DENGMPPSAGTSSRVRPTKYLVFSNTVHGLGNHMAGMMSAFVMSIATERVFLHDWVEP
MSTRG.51532.1    RGNDAFSPAQAEGSLRVQPTKYLVFVNTVHGLGNHMAGMMSAFVLSIVTERVFLHDWVEP
                        #####################################################


                        310       320       330       340       350       360
                 =========+=========+=========+=========+=========+=========+
Ec-12_003690     RAQVAARYNDIFVAPFTPWDTSAATGTQVSGPEDLLTESGGLGGRPANQAAAGSSGERWA
MSTRG.51532.1    RGQVAARYDELFVPPFEPWDTSAAPGSLISGPDGLLP-------AAAKVQPSKSVGQSWV
                 ####################################          ##############


                        370       380       390       400       410       420
                 =========+=========+=========+=========+=========+=========+
Ec-12_003690     KPPEMSSAEEKERCTLDFKPTGGVQKGHFNAVFLKKRGQDPRPKCPVLYVSSNQFLVEFM
MSTRG.51532.1    KIPEISSEKESKRCRLEFTPSGGVTKGHFNDVFLRKRGQEPRPDCPVLFVFTNQFLVEIM
                 ############################################################


                        430       440       450       460       470       480
                 =========+=========+=========+=========+=========+=========+
Ec-12_003690     SSPAFGSSKVGGTLISEWLRPGRASQTLFPGLFRPVAAVRQRIADESARLFPVEGQTIAA
MSTRG.51532.1    ASPAYGSSEVGGVHISEWLRAGRASNTLFHGLFRAVAPVRQRVEDEALRLFPNDGQTMAA
                 ############################################################


                        490       500       510       520       530       540
                 =========+=========+=========+=========+=========+=========+
Ec-12_003690     HARSFHLGSVSRNNKFVGCLQQLALSKNISTVLLVTDMENFEQFAASKLRPRGVNVVTMN
MSTRG.51532.1    HARSFFLGSIPRNNKFVGCLQQLALSKNISTVLLASDMNDFDKLVASKLKPHGINVVMMK
                 ############################################################


                        550       560       570       580       590       600
                 =========+=========+=========+=========+=========+=========+
Ec-12_003690     GVERSGEGQKMRNSLQKIQLALTEMMLVGNADIAVTSPRSSYSALAAARGGSSTQRYGGI
MSTRG.51532.1    GVQRSGEGAKMRNTLGKIQLALTEMMLVGKADIVVTSPRSSFSALAAASGGESTERFGGI
                 ############################################################


                        610       620       630       640
                 =========+=========+=========+=========+========
Ec-12_003690     SCTEQVNEPEFHCLECAKNEWKVDQVKNPTEWDKVYHLVESNRARCKG
MSTRG.51532.1    SCKSLLNEPEFHSLECAKHEWHVEQVGNPKQWDEVYHVVGSNSARCKG
                 ################################################
```

```
Parameters used
Minimum Number Of Sequences For A Conserved Position: 2
Minimum Number Of Sequences For A Flanking Position: 2
Maximum Number Of Contiguous Nonconserved Positions: 8
Minimum Length Of A Block: 10
Allowed Gap Positions: None
Use Similarity Matrices: Yes
```

```
Flank positions of the 5 selected block(s)
Flanks: [87  96]  [132  150]  [209  239]  [248  336]  [347  648]  

New number of positions in Ec-12_003690.aa.fasta.trans.aa_ali.fasta-gb:  451  (69% of the original 648 positions)
```
